# Targeting the YXXΦ Motifs of the SARS Coronaviruses 1 and 2 ORF3a Peptides by In Silico Analysis to Predict Novel Virus—Host Interactions

**DOI:** 10.3390/biom12081052

**Published:** 2022-07-29

**Authors:** Athanassios Kakkanas, Eirini Karamichali, Efthymia Ioanna Koufogeorgou, Stathis D. Kotsakis, Urania Georgopoulou, Pelagia Foka

**Affiliations:** 1 Laboratory of Molecular Virology, Hellenic Pasteur Institute, 115-21 Athens, Greece; thanos@pasteur.gr (A.K.); eirinik@pasteur.gr (E.K.); ekoufogeorgou@pasteur.gr (E.I.K.); uraniag@pasteur.gr (U.G.); 2 Laboratory of Bacteriology, Hellenic Pasteur Institute, 115-21 Athens, Greece; skotsakis@pasteur.gr

**Keywords:** SARS-CoV, SARS-CoV-2, ORF3a, YXXΦ motif, post-translational modifications, immune response

## Abstract

The emerging SARS-CoV and SARS-CoV-2 belong to the family of “common cold” RNA coronaviruses, and they are responsible for the 2003 epidemic and the current pandemic with over 6.3 M deaths worldwide. The ORF3a gene is conserved in both viruses and codes for the accessory protein ORF3a, with unclear functions, possibly related to viral virulence and pathogenesis. The tyrosine-based YXXΦ motif (Φ: bulky hydrophobic residue—L/I/M/V/F) was originally discovered to mediate clathrin-dependent endocytosis of membrane-spanning proteins. Many viruses employ the YXXΦ motif to achieve efficient receptor-guided internalisation in host cells, maintain the structural integrity of their capsids and enhance viral replication. Importantly, this motif has been recently identified on the ORF3a proteins of SARS-CoV and SARS-CoV-2. Given that the ORF3a aa sequence is not fully conserved between the two SARS viruses, we aimed to map in silico structural differences and putative sequence-driven alterations of regulatory elements within and adjacently to the YXXΦ motifs that could predict variations in ORF3a functions. Using robust bioinformatics tools, we investigated the presence of relevant post-translational modifications and the YXXΦ motif involvement in protein-protein interactions. Our study suggests that the predicted YXXΦ-related features may confer specific—yet to be discovered—functions to ORF3a proteins, significant to the new virus and related to enhanced propagation, host immune regulation and virulence.

## 1. Introduction

Beta coronaviruses (CoV) have been responsible for three important outbreaks over the last 20 years: Severe Acute Respiratory Syndrome Coronavirus (SARS-CoV) epidemic in 2003, Middle East Respiratory Syndrome-Coronavirus (MERS-CoV) epidemic in 2012 and the ongoing Severe Acute Respiratory Syndrome Coronavirus-2 (SARS-CoV-2) pandemic, officially declared as such in March 2020. To date, all three viruses constitute a serious health concern [1]. SARS-CoV and SARS-CoV-2 affect humans with a wide range of mild-to-severe symptoms. Notably, SARS-CoV-2 may cause an asymptomatic sub-clinical infection, or mild “flu-like” symptoms concerning the upper respiratory system, or severe lung and heart complications that can be fatal, depending on the individual afflicted [2]. SARS-CoV and SARS-CoV-2 have a fatality rate of 9.7% and 2.3%, respectively [3]. MERS-CoV emerged approximately a decade after SARS-CoV. It is mainly transmitted by dromedary camels and close human-to-human contacts [4] and causes acute pneumonia [5] with a high fatality rate of 35% [6].

MERS-CoV possesses a genome of 30.1 kb, while SARS-CoV and SARS-CoV-2 have genome sizes of 27.9 kb and 29.9 kb, respectively. MERS-CoV encodes ten proteins; two replicases (ORF1ab and ORF1a), the four structural proteins Envelope (E), Membrane (M), Nucleocapsid (N) and Spike (S) [7], which compose the enveloped viral capsid [8] and four non-structural proteins, also called accessory proteins, ORF3, ORF4a, ORF4b and ORF5 [7]. SARS-CoV and SARS-CoV-2 genomes encode two replicases (ORF1a and ORF1b), four capsid/envelope proteins (E, M, N, S), 15 or 16 non-structural proteins and several accessory proteins, ORF3a, 3b, 6, 7a, 7b, 8a, 8b and 9b for SARS-CoV [9] and ORF3a, 3b, 3c, 3d, 6, 7a, 7b, 8, 9b, 9c and 10 for SARS-CoV-2 [10], respectively.

SARS-CoV-ORF3a and SARS-CoV-2-ORF3a proteins (274–275 aa) have been identified to be viroporins [11,12]. The term refers to a group of small viral proteins that share several common functions [13,14]. Importantly, viroporins can alter membrane permeability and oligomerise to form a hydrophilic pore that allows ion transport across host-cell membranes, as shown for the Influenza A Virus (IAV) M2 protein [15], the Hepatitis C Virus (HCV) p7 protein [16] the Coxsakie Virus 2B [17] proteins and others. They have also been involved in several viral processes, including replication, virion assembly and virus entry and egress [18,19,20]. The ORF3a proteins of β-coronaviruses play a key-role in viral egress via lysosomal trafficking [21,22] and they are implicated in viral infectivity and pathogenesis [13,23]. It has been demonstrated that SARS-CoV-ORF3a induces apoptosis in host cells [24], promotes membrane rearrangements and cell death [25] and inhibits type-I IFN signalling and antiviral defences [26]. Very recent data suggest that SARS-CoV-2-ORF3a also exerts a proapoptotic activity against host cells, albeit relatively weaker than that of the SARS-CoV-ORF3a protein [27], and shares a similar role with the latter in regulating viral egress [13]. It has also been shown to be a negative regulator of host cellular autophagy, thereby helping the virus to evade destruction in the lysosomes [28].

YXXΦ is a tyrosine-based motif (where X can be any residue and Φ is a residue with a bulky hydrophobic side chain) that directs the clathrin-dependent endocytosis of membrane-spanning proteins into clathrin-coated vesicles (CCVs) by clathrin adaptor proteins (APs) [29,30]. The YXXΦ motifs also constitute part of a different type of signalling motifs, ones that regulate immune responses through activating and inhibitory receptors on the surface of immune and other cells. These interactions depend on two conserved motifs that are found in the intracellular domain of many signalling proteins. They are called “ITAMs” (YXXL/I(X6-12) YXXL/I) and “ITIMs” (I/V/L/S)-X-Y-X-X-(L/V), for immunoreceptor tyrosine-based activation (or inhibitory) motifs, and they are regulated through tyrosine phosphorylation [31]. Some viruses have adopted the YXXΦ motif into their proteins and use it for immune regulation [32], sorting purposes [33,34] or other yet-to-be-identified functions. To that end, previous data from our laboratory have revealed that the YXXΦ motif of HCV core nucleocapsid protein is critical for non-enveloped capsid-like particles’ (HCVne) architectural integrity and form. Furthermore, this motif is crucial for the signalling events that follow HCVne clathrin-mediated endocytosis, AP-2 clathrin adaptor protein induction and the HCVne trafficking to the lipid droplets (LDs) that finally enhance viral replication [35].

Interestingly, the cytoplasmic domain of the SARS-CoV-ORF3a contains an experimentally-verified YXXΦ motif at position 160–163 aa [9,36], although others may exist. This YXXΦ has been previously reported to be responsible for lysosomal targeting, endocytosis [37] and trafficking of ORF3a protein from the Golgi apparatus towards the plasma membrane [9,36]. Notably, the motif has been shown to be conserved in SARS-CoV-2-ORF3a [9,36,38], while there is no information available concerning the presence of such a motif in the ORF3 of MERS-CoV.

Considering that the ORF3a aa sequence is not absolutely conserved between SARS-CoV and SARS-CoV-2, this study aimed to map putative YXXΦ motifs through in silico analysis. Then, we identified structural differences and sequence-driven changes of regulatory elements within and adjacently to the YXXΦ motifs that could potentially attribute gain- or loss-of-function properties, significant for virulence, pathogenesis and host immune regulation, to the ORF3a proteins.

## 2. Materials and Methods

### 2.1. Viral Sequences, Alignment and Motif Analyses

The viral sequences of MERS-CoV-ORF3, SARS-CoV-ORF3a and SARS-CoV-2-ORF3a were retrieved from the GenBank with the following accession numbers: YP_009047205.1 (MERS-CoV) [39], AY274119.3 (isolate Tor2) [40] and YP_009724391.1 (SARS-CoV-2 isolate Wuhan-Hu-1) [41], respectively.

Protein alignment was performed using the multiple sequence alignment Clustal Omega web server (https://www.ebi.ac.uk/Tools/msa/clustalo/, accessed on 21 July 2022) [42]. The determination of the ΥΧΧΦ [M/L/I/V/F] and the di-leucine motifs was carried out using the Eukaryotic Linear Motif (ELM) prediction web server (http://elm.eu.org/index.html, accessed on 21 July 2022) [43]. The non-canonical and reverse ΥΧΧΦ motifs were mapped onto the viral sequences manually, based on existing literature [44,45].

### 2.2. Analysis of Structural Features

The topology of transmembrane, extracellular and intracellular regions was performed using the Protter web server (http://wlab.ethz.ch/protter/, accessed on 21 July 2022), which predicts transmembrane regions, using both annotated sequence features and experimental proteomic data [46]. Prediction of secondary structure features and the location of α-helices, β-sheets and coil segments of SARS-CoV-ORF3a and SARS-CoV-2-ORF3a was determined with the use of DeepGSH web server (http://deepgsh.omicsbio.info/index.php, accessed on 21 July 2022) [47].

Internal disordered regions of SARS-CoV-ORF3a and SARS-CoV-2-ORF3a were identified using the server PONDR-XL1_XT (Predictor of Natural Disordered Regions) (http://www.pondr.com/, accessed on 21 July 2022) with the XL1_XT analysis predictor [48]. Changes in protein stability upon in silico mutagenesis were determined by the DynaMut web server (http://biosig.unimelb.edu.au/dynamut/, accessed on 21 July 2022) developed to analyse and visualise protein dynamics of the impact of mutations from vibrational entropy changes [49].

For the modelled structures of the ORF3a proteins, the atomic coordinates of the SARS-CoV-2-ORF3a were extracted from the latest version of the Cryo-EM structure of the protein (PDB ID: 7KJR; chains A and B; resolution: 2.08 Å; residues 40–238) [50]. The missing loop, containing the residues T_175_TSPIS, was built through homology modelling using the MODELLER v9.22 package and the 7KJR structure as template. A three-dimensional model of the SARS-CoV-ORF3a (74% identical to the 40–238 portion of SARS-CoV-2-ORF3a) dimer was built through homology modelling as above using extended refinement of the generated models (*n* = 20) through a detailed variable target function method (VTFM) and simulated annealing as implemented in MODELLER [51]. The models exhibiting the best probability density function scores were energy minimised in vacuo in GROMACS 2016 [52] using the *steepest descents* and *conjugated gradient* algorithms until *F*_max_ < 10 KJ∙mol^−1^∙nm^−1^ with atomic parameters being those of the AMBER99SB-ILDN force field. The minimised structures of the SARS-CoV-ORF3a and SARS-CoV2-ORF3a were superimposed using the backbone atoms for the root mean square fit (backbone RMSD = 0.5 Å).

### 2.3. In Silico Analysis of Post-Translational Modifications (PTMs)

Tyr, Ser and Thr phosphorylation prediction was performed with the NetPhos-3.1 server (https://services.healthtech.dtu.dk/service.php?NetPhos-3.1, accessed on 21 July 2022) [53].

Lys ubiquitination was predicted with the use of the web server BDM-PUB (Prediction of Ubiquitination sites with Bayesian Discriminant Method) (http://bdmpub.biocuckoo.org/prediction.php, accessed on 21 July 2022) [54].

Prediction of Lys succinylated residues was carried out with the iSuc-PseAAC web server (http://app.aporc.org/iSuc-PseAAC/, accessed on 21 July 2022) [55]. 

Lys methylation prediction was performed using the web server iMethyl-PseAAC (http://www.jci-bioinfo.cn/iMethyl-PseAAC, accessed on 21 July 2022) [56], while prediction of acetylation on internal lysines was done with the use of the PAIL web server (http://bdmpail.biocuckoo.org/prediction.php, accessed on 21 July 2022), an acetylation prediction tool with high accuracy [57].

The probability of Tyr nitration was calculated using the iNitro-Tyr web server, (http://app.aporc.org/iNitro-Tyr/index.html, accessed on 21 July 2022) [58], while S-Nitrosylation and S-Glycosylation prediction of Cys residues was carried out by the pCysMod web server (http://pcysmod.omicsbio.info/webserver.php, accessed on 21 July 2022) [59]. 

Prediction of N-linked glycosylation on Asp residues was performed with the GlycoEP web server, using the Composition Profile of Patterns (CPP)-based prediction option (https://webs.iiitd.edu.in/raghava/glycoep/submit.html, accessed on 21 July 2022). This server specialises in the prediction of N-, O- and C-linked glycosides [60].

S-glutathionylation prediction was performed with the DeepGSH web server (http://deepgsh.omicsbio.info/action.php, accessed on 21 July 2022), suitable for the identification of S-glutathionylation sites in proteins [61].

N-myristoylation site prediction was carried out using the PROSITE web server (https://prosite.expasy.org/scanprosite/, accessed on 21 July 2022) [62].

Finally, Tyr sulfation probability was predicted using the GPS-TSP web server (http://tsp.biocuckoo.org/index.php, accessed on 21 July 2022) [63].

### 2.4. Immunoreactive Epitope Prediction Analysis

Prediction of regions within the SARS-CoV-ORF3a and SARS-CoV-2-ORF3a ΥΧΧΦ motifs and ΥΧΧΦ-like tetrapeptides that are likely to cause a B-cell response by acting as linear antigenic epitopes, was performed using the Immune Epitope Database (IEDB) Analysis Resource web server (http://tools.iedb.org/bcell/, accessed on 21 July 2022). This online tool predicts B-cell epitopes, using the Chou and Fasman beta turn prediction method [64]. For prediction of discontinuous conformational antigenic epitopes, we used the Epitope3D online tool [65]. The existence of putative ITAM/ITIM motifs was investigated by ELM protein analysis [43].

## 3. Results

### 3.1. Topology of SARS-CoV and SARS-CoV-2 YXXΦ Motifs

**Figure 1:**
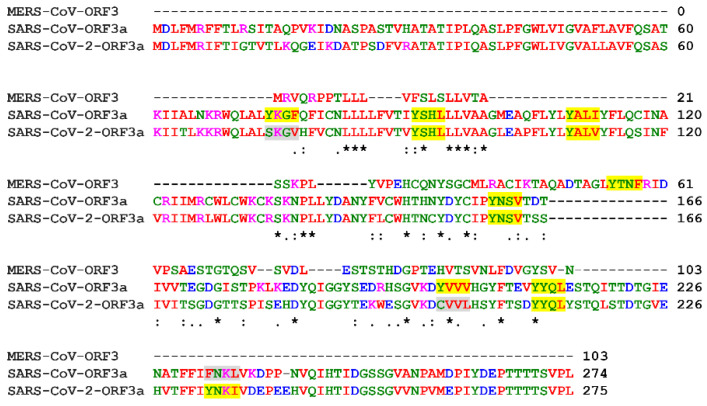
Alignment of MERS-CoV-ORF3, SARS-CoV-ORF3a and SARS-CoV-2-ORF3a sequences. YΧΧΦ motifs are represented in yellow and the YXXΦ-like tetrapeptides at the same position, in grey. Stars: amino acid sequence similarity; colons: amino acid sequence difference.

SARS-CoV-ORF3a shares 73% homology with SARS-CoV-2-ORF3a and only 50% similarity with MERS-CoV-ORF3 [27]. Therefore, all subsequent studies will be focused on the ORF3a proteins of the two SARS viruses, SARS-CoV and SARS-CoV-2. Along SARS-CoV-ORF3a and SARS-CoV-2-ORF3a sequences, there are putative canonical YXXΦ motifs or YXXΦ-like tetrapeptides, where the motif is “disrupted” due to amino acid substitution(s) (Figure 1).

In the present study, ΥΧΧΦ [M/L/I/V/F] motifs located within the SARS-CoV-ORF3a and SARS-CoV2-ORFa were identified by ELM prediction analysis. SARS-CoV-ORF3a protein has six putative ΥΧΧΦ motifs, while SARS-CoV-2-ORF3a has five. Both ORF3a proteins contain four identical ΥΧΧΦ motifs located in the same positions, named herein as first, second, third and fourth. Additionally, SARS-CoV-ORF3a possesses two unique YXXΦ motifs, located in positions (74–77 aa) and (200–203 aa), which will be named from now on as “SCoV-upstream” and “SCoV-extra”, respectively. In SARS-CoV-2-ORF3a these upstream and extra YXXΦ motifs contain residual substitutions and have been converted to SKGV (74–77 aa) and CVVL (200–203 aa) YXXΦ-like tetrapeptides. In SARS-CoV-2-ORF3a, there is a unique YXXΦ motif, YNKI (233–236 aa), named herein as “S2CoV-downstream”. In SARS-CoV-ORF3a, the tetrapeptide FNKL (233–236 aa) replaces the downstream YXXΦ motif of SARS-CoV-2-ORF3a (Table 1).

**Figure 2:**
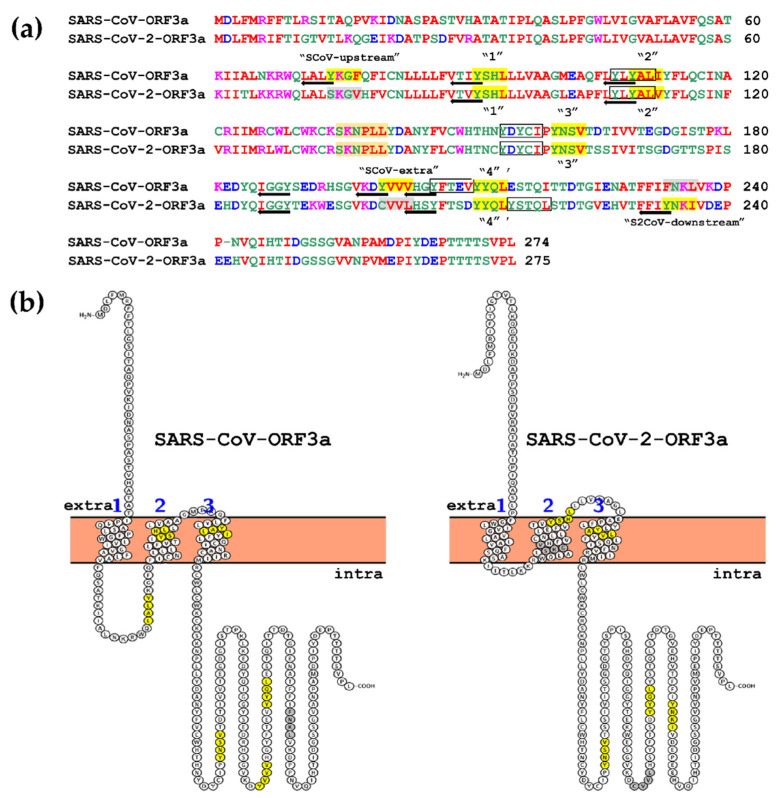
Topology of SARS-CoV-ORF3a and SARS-CoV-2-ORF3a YXXΦ motifs. (**a**) Schematic representation of the location of YXXΦ motifs and YXXΦ–like tetrapeptides within the SARS-CoV-ORF3a and SARS-CoV-2-ORF3a proteins is shown in yellow and grey, respectively. The non-canonical YXXXΦ motifs, the reverse ΦXXY motifs and the di-leucine motifs are represented in black frames, with black arrows and in light orange frames, respectively. Missing amino acids are denoted by a dash. (**b**) Schematic representation of the TM regions of SARS-CoV-ORF3a and SARS-CoV-2-ORF3a and indication of the topology of canonical ΥΧΧΦ motifs and ΥΧΧΦ-like tetrapeptides. YΧΧΦ motifs are presented in yellow circles and ΥΧΧΦ-like tetrapeptides, in grey circles. Extra: Extracellular; Intra: Intracellular.

In addition to the canonical YΧΧΦ motifs, other similar motifs have also been described in the literature, such as the non-canonical YXXXΦ motifs [45], the reverse ΦXXY motifs and the di-leucine peptides, all of which have been proposed to act as internalisation motifs [44,66]. The identification of the non-canonical ΥΧΧXΦ[M/L/I/V/F] and the reverse Φ[M/L/I/V/F]ΧΧY motifs on the sequence of ORF3a proteins was performed manually while the di-leucine motifs were predicted by ELM analyses (Table S1). All the ΥXXΦ motifs and the ΥXXΦ-like tetrapeptides, the non-canonical ΥΧΧXΦ, the reverse ΦΧΧY motifs, as well as the di-leucine motifs of SARS-CoV-ORF3a and SARS-CoV-2-ORF3a are schematically presented in Figure 2a. As previously reported, ORF3a proteins contain three transmembrane α-helices at the N terminus and a C-terminal cytoplasmic domain of 150 aa [67]. For the visualisation of the topology of transmembrane (TM), extracellular and intracellular regions, as well as the canonical YXXΦ motifs and YXXΦ-like tetrapeptides, we used the Protter online server (Figure 2b).

The predicted TM helices topology within the SARS-CoV-ORF3a and SARS-CoV-2-ORF3a proteins is shown in Table S2.

### 3.2. Structural Features and Disordered Domains in SARS-CoV-ORF3a and SARS-CoV-2-ORF3a Proteins

**Figure 3:**
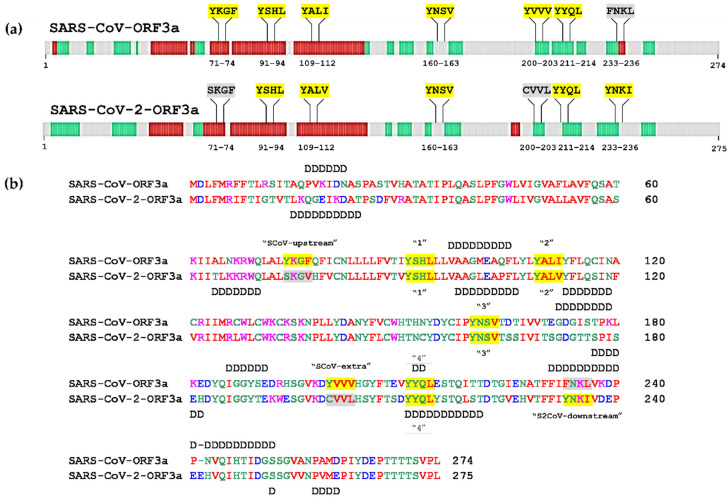
Structural prediction analysis of SARS-CoV-ORF3a and SARS-CoV-2-ORF3a proteins. (**a**) Schematic representation of secondary structure of SARS-CoV-ORF3a and SARS-CoV-2-ORF3a proteins. Red boxes represent the α-helix structures, green boxes the β-sheet structures and gray boxes depict coil segments. (**b**) Prediction of the internal disordered regions in SARS-CoV-ORF3a and SARS-CoV2-ORF3a proteins. The disordered residues are noted with the letter “D”. YXXΦ motifs are presented in yellow and the YXXΦ-like tetrapeptides in grey.

Structural prediction analyses carried out with the Deepgsh server [47] revealed subtle differences between SARS-CoV-ORF3a and SARS-CoV-2-ORF3a, concerning α-helices, β-sheets and coil segments, as depicted in Table S3. The presence of ΥΧΧΦ motifs and ΥΧΧΦ-like tetrapeptides was mapped on the structural features of both proteins. Evidently, the S2CoV-downstream ΥΧΧΦ motif (233–236 aa) was found lying between a β-sheet and coil segment, while the corresponding ΥΧΧΦ-like tetrapeptide of SARS-CoV-ORF3a was situated on a short α-helix (Figure 3a). No other differences were observed.

Intrinsically-disordered regions are involved in several cellular processes, such as signalling and gene transcription [68]. These regions are considered more “flexible” than the rigid regions, such as alpha helices and beta sheets. Therefore they can be subjected to post-translational modifications (PTMs) and interact with many cellular partners in a promiscuous way [69]. Such domains offer high flexibility to viral proteins and provide a quick adaptation to the host environment, survival and evasion from the defence mechanism of the host (reviewed in Mishra et al., 2020) [70]. Prediction analysis with the PONDR-XL1_XT predictor [48] revealed that only the fourth YXXΦ motif of both viral proteins and the SCoV-upstream YXXΦ motif-like tetrapeptide are situated within disordered regions (Figure 3b).

### 3.3. Post-Translational Modifications within Motifs

PTMs are a heterogeneous group of chemical reactions that occur on the side chains of a protein residue and ultimately affect both its structure and function. The prediction of PTMs is considered a very useful tool for the detection and understanding of protein-protein interactions [71]. In coronaviruses, protein PTMs contribute to viral pathogenesis, enhance antigenicity and regulate virulence [72]. Because RNA viruses do not possess the necessary enzymes for introducing PTMs to their proteins, they utilise the host PTM machinery to complete the viral life cycle [43].

#### 3.3.1. Phosphorylation

**Figure 4:**
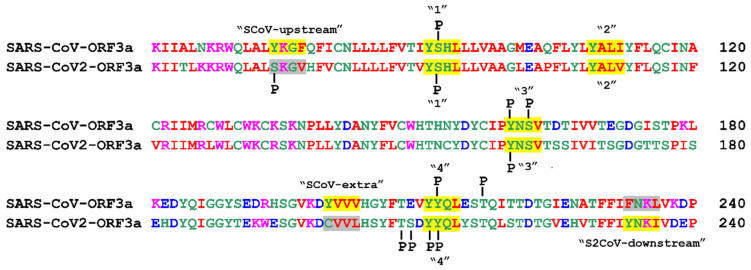
Prediction of putative phosphorylated residues within the YXXΦ motifs, the YXXΦ-like tetrapeptides and their adjacent sequences in SARS-CoV-ORF3a and SARS-CoV-2-ORF3a proteins. The YXXΦ motifs are shown in yellow and the YXXΦ-like tetrapeptides in grey. P: Phosphorylation.

Phosphorylation is the most common and well-studied post-translational modification [73]. It is catalysed by kinases that covalently attach a phosphoryl group to the side chain of Ser, Thr and Tyr residues in a reversible way, irrespectively of the position of the amino acid on a specific structural feature [74]. Prediction of putative phosphorylated amino acid residues within the YXXΦ motifs, the YXXΦ-like tetrapeptides and adjacent sequences for SARS-CoV-ORF3a and SARS-CoV-2-ORF3a proteins with the NetPhos3.1 prediction server [53], revealed the following: The Ser74 residue of the SKGV (74–77 aa) tetrapeptide in SARS-CoV-2-ORF3a, can be phosphorylated. On the contrary, Tyr74 of the SCoV-upstream YXXΦ (74–77 aa) motif was not predicted to be phosphorylated. Ser92 of the first ΥΧΧΦ (91–94 aa) motif may be phosphorylated in both SARS-CoV-ORF3a and SARS-CoV-2-ORF3a. Another phosphorylation event may occur at the Tyr160 residue of the third YΧΧΦ (160–163 aa) motif, which is located in a coil segment (Figure 3a), in both ORF3a proteins. Ser162 of the third YNSV (160–163 aa) motif of SARS-CoV-ORF3a can also be phosphorylated, while the equivalent Ser162 in SARS-CoV-2-ORF3a may not. Tyr211 located in the fourth YΧΧΦ (211–214 aa) motif of SARS-CoV-2-ORF3a was predicted to be phosphorylated, while the equivalent Tyr211 of SARS-CoV-ORF3a was not. Tyr212 of the fourth YYQL motif in both ORF3a proteins also exhibited high chances of being phosphorylated. The adjacent Thr208 and Ser209 of SARS-CoV-2-ORF3a may also be phosphorylated, according to the same analysis. Finally, only the Thr216 of SARS-CoV-ORF3a was predicted to be phosphorylated (Figure 4). All the NetPhos3.1 prediction scores, as well as the kinases catalysing each putative phosphorylation event, are displayed in Table S4.

#### 3.3.2. Ubiquitination

**Figure 5:**
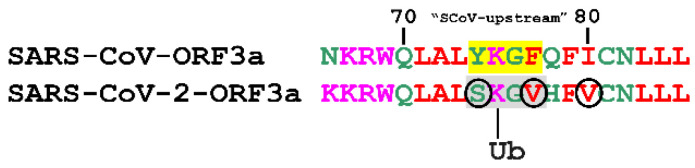
Prediction of a putative ubiquitination PTM on a SARS-CoV2-ORF3a YXXΦ-like tetrapeptide (in grey). The corresponding YXXΦ motif in SARS-CoV-ORF3a is depicted in yellow. The residues indicated in black circles promote Lys ubiquitination. Ub: Ubiquitination.

Ubiquitination refers to the reversible enzymatic modification where the polypeptide ubiquitin uses its C-terminus Gly76 residue to covalently bind lysines in the protein substrate [75]. Ubiquitination is catalysed by a complex network of enzymes. It can take place in all 20 amino acids, but it mainly occurs in Lys residues [71]. Using the ubiquitin prediction web-server BDM-PUB, we found that SARS-CoV-ORF3a and SARS-CoV-2-ORF3a contain several putative ubiquitination sites, listed in Table S5. Interestingly, the Lys75 residue of SARS-CoV-2-ORF3a protein, which is located in the upstream-like YXXΦ-like tetrapeptide (74–77 aa) of SARS-CoV-2, was predicted to be ubiquitinated with an excellent score, in contrast to the corresponding Lys75 of ScoV-upstream motif (Figure 5). 

The ubiquitination of Lys75 could be enhanced by the presence of neighbouring residues Ser74 (at −1 position) and Val (at +2 and +5 positions), which may facilitate the formation of this PTM. Lys75 of SARS-CoV-ORF3a, might not be a good candidate for ubiquitination, due to the presence of the Tyr74 (at −1 position), Phe77 (at +2 position) and Ile80 (at +5 position). This hypothesis was confirmed by in silico mutagenesis on SARS-CoV-2-ORF3a sequences, using the abovementioned server. A S74Y mutation could prove deleterious for ubiquitination, while a V77P and a V80I mutation could greatly reduce the ubiquitin attachment probability. These results are presented in Table S6. Finally, Lys235 of SARS-CoV-ORF3a, located in the downstream-like FNKL tetrapeptide (YXXΦ-like motif, 233–235 aa) held a very low chance of being ubiquitinated due to its marginally positive score (Table S5).

#### 3.3.3. Succinylation

**Figure 6:**
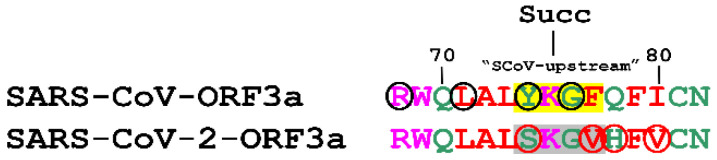
Prediction of a putative succinylation PTM on a SARS-CoV-ORF3a YXXΦ motif (in yellow). The residues indicated in black circles promote Lys succinylation. The residues in red circles inhibit Lys succinylation of the YXXΦ–like tetrapeptide of SARS-CoV-2-ORF3a protein (in grey). Succ: Succinylation.

Lysine succinylation is a reversible PTM, where a succinyl group (-CO-CH_2_-CH_2_-COOH) is bound to a Lys residue of the targeted protein. Succinylation can alter the charge of the lysine residue from +1 to −1, under certain physiological pH conditions [76]. Predictions by the iSuc-PseAAC server [55], revealed that Lys75 of SARS-CoV-ORF3a can be succinylated, whereas the corresponding Lys75 of SARS-CoV-ORF3a cannot. The in silico residue substitution we performed showed that Ser75, Vall77, His78 and Val80 residues of SARS-CoV-ORF3a, facilitated Lys75 succinylation. Conversely, the combination of Val77 and His78 residues, just after Lys75, dramatically reduced this probability, proving that this combination inhibits K75 succinylation in SARS-CoV-2-ORF3a (Figure 6).

#### 3.3.4. Methylation

Upon protein methylation, the NH_2_ group of lysine residues can accept up to three methyl groups in reactions catalysed by the family of protein lysine methyltransferase (PKMT) enzymes [77]. Although Lys methylation has been widely associated with histone modifications and epigenetic changes, recent studies have implicated this PTM in the modification of non-histone proteins [78].

**Figure 7:**
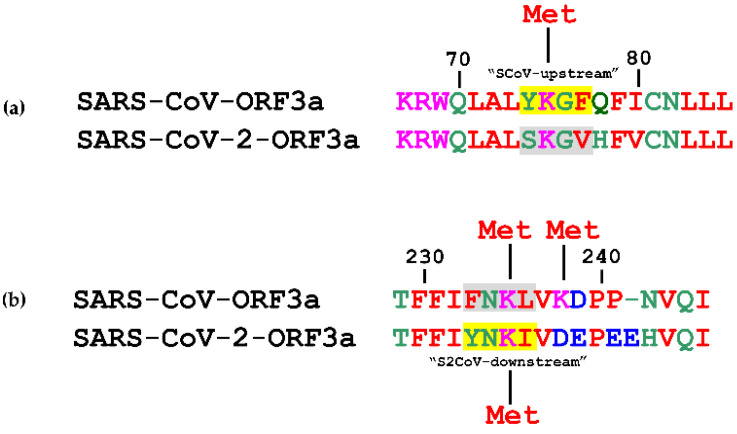
Prediction of putative Lys methylation on (**a**) Lys75 and (**b**) on Lys235 of SARS-CoV-ORF3a and SARS-CoV-2-ORF3a YXXΦ motifs (in yellow) and YXXΦ-like tetrapeptides (in grey). Missing amino acids are denoted by a dash. Met: Methylation.

In silico analyses of Lys methylation with iMethyl-PseAAC [56], showed that the Lys75 located in the SCoV-upstream YKGF (74–77 aa) motif was predicted to be methylated, whereas the corresponding Lys75 of SARS-CoV-2-ORF3a was not (Figure 7a). In SARS-CoV-ORF3a, the probability for Lys75 methylation could be enhanced by the presence of Gly78 (at +3 position) and Phe79 (at +4 position). Lys235 in both strains (S2CoV-downstream YNKI motif (233–236 aa) motif and FNKL (233–236 aa) YXXΦ-like tetrapeptide, respectively), as well as the adjacent Lys238 of SARS-CoV-ORF3a, also appeared methylated (Figure 7b).

In silico analysis of lysine flanking residues showed that methylation in Lys235 of SARS-CoV-ORF3a could be enhanced by the presence of Leu236 (at +1 position) and Asp239 (at +4 position) residues. Similarly, Leu236 (at +1 position), Ap239 (at +3 position) and Glu239 (at +4 position) may play a crucial role in the methylation of Lys235 of SARS-CoV-2-ORF3a. Finally, in SARS-CoV-ORF3a, Lys238 methylation may be enhanced by the presence of Asp239 (at +1 position) and Asn242 (at +4 position) [77]. Putative methylated residues on the YXXΦ motifs of SARS-CoV-ORF3a and SARS-CoV-2 ORF3a are shown in Table S7.

#### 3.3.5. Acetylation

**Figure 8:**
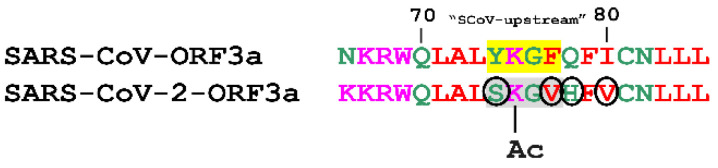
Prediction of an acetylation PTM in a YXXΦ-like tetrapeptide (in grey) of SARS-CoV-2-ORF3a. The corresponding YXXΦ motif of SARS-CoV-ORF3a is shown in yellow. Black circled residues are predicted to promote acetylation. Ac: Acetylation.

Lys acetylation is a common, reversible PTM and entails the transfer of an acetyl group from acetyl coenzyme A to the side chain of a Lys residue at the ε-position of the lysine within a protein, a process that leads to neutralisation of the position’s positive electrostatic charge [79]. With the use of the PAIL server [57], it was shown that Lys75 in the YXXΦ-like tetrapeptide of SARS-CoV-2-ORF3a can be acetylated, in contrast to the Lys75 residue, which is placed within the SCoV-upstream YXXΦ-motif (Figure 8). In silico mutagenesis analyses revealed that Lys75 acetylation is enhanced mainly by the presence of Ser74 (at −1 position) and to a lesser extent by Val77 (at +2 position), His78 (at +3 position) and Val80 (at +5 position). The full results of the in silico prediction of the internal acetylated Lys residues in SARS-CoV-ORF3a and SARS-CoV-2-ORF3a are depicted in Table S8.

#### 3.3.6. Nitration

**Figure 9:**
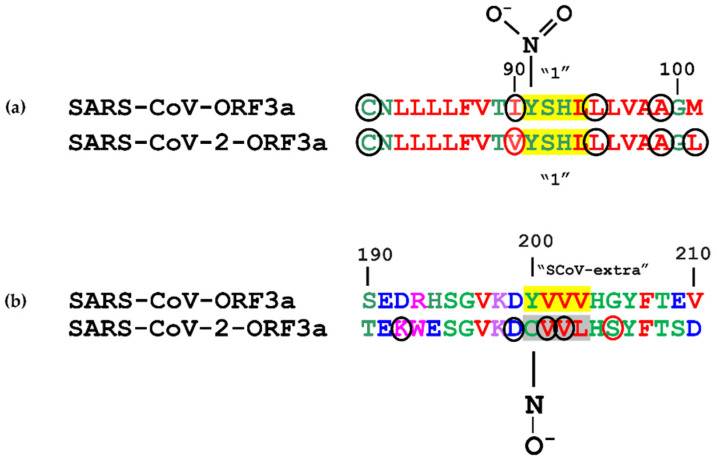
Prediction of (**a**) putative Tyr nitration and (**b**) an S-nitrosylation event on SARS-CoV-ORF3a and SARS-CoV-2-ORF3a YXXΦ motifs (in yellow). Grey denotes a YXXΦ-like tetrapeptide. The residues indicated in black circles promote Tyr and Cys PTMs, while the red circles have the opposite effect.

Tyrosine nitration is a reversible PTM where, upon transient oxidation, a free radical ^•^NO_2_ (or ^•^NO or ONOO^+^) group covalently binds to the Tyr phenolic ring, thereby converting the Tyr to 3-nitro-tyrosine [80]. Both SARS-CoV-ORF3a and SARS-CoV-2-ORF3a proteins possess 17 Tyr residues and contain the first YXXΦ (91–94 aa) motif, which is situated at the C-terminal region of the second α-transmembrane helix, in both strains. In silico analyses with iNitro-Tyr server [58], showed that Tyr91 of the first motif of SARS-CoV-ORF3a is nitrated. No other tyrosines involved in the formation of the YXXΦ motifs of both ORF3a strains were predicted to be nitrated. Herein, the suggested residues that enhance the probability of a tyrosine nitration are Cys (at −10 position), Leu (at +4 position) and Ala (at +8 position) for both SARS-CoV-ORF3a and SARS-CoV-2-ORF3a. Tyr91 of SARS-CoV-2-ORF3a may have lost the potential for nitration due to the existence of a Val residue (at −1 position) next to Tyr91, that strongly inhibits nitration, despite the presence of another nitration enhancing residue (Leu at position +10) [81] (Figure 9a).

SARS-CoV-ORF3a possesses a Tyr, at position 200, which enables the formation of the SCoV-extra YXXΦ motif, the YVVV tetrapeptide at position 200–203. This ScoV-extra YXXΦ motif is abolished in SARS-CoV-2-ORF3a protein because of an amino acid substitution at position 200 (Y200C), which leads to the formation of a CVVL tetrapeptide. Both tetrapeptides are located on a short β-sheet (Figure 3a), denoting that the YVVV→CVVL (200–203 aa) alteration does not influence the secondary structure of the protein much. Analyses with the Dynamut server [49] showed that a C200Y mutation could stabilise the protein (ΔG: 0.652 kcal/mol), resulting in rigidification of the β-sheet and the L203V mutation could slightly destabilise it, causing a gain in flexibility. Notably, in silico analyses with the server of pCysMod [59] specific for predicting PTMs of cysteine residues suggested that the C200 residue of SARS-CoV-2-ORF3a is S-nitrosylated (Figure 9b). Our data suggest that the Asp (at −1 position), the Lys (at −2 position), the Val (at +1 and +2 positions) and the distal Lys (at −8 position) increase the chances of an Cys200 nitrosylation, while the Ser (at +5 position) slightly restrict PTM occurrence [82]. Full Tyr nitration prediction results appear in Table S9, while Table S10 presents data on C200 S-nitrosylation.

#### 3.3.7. N-Glycosylation

N-Glycosylation is the covalent attachment of an oligosaccharide, such as N-glycans, to the N atom of an Asn residue that lies within a typical glycosylation motif N-X-S/T (where X: any aa except Pro) [83]. Furthermore, there are atypical glycosylation motifs known as NXV (where X: any aa except Pro) [84]. Alternatively, an oligosaccharide could be linked to the O-atom of Ser/Thr or to the S-atom of Cys, conferring O-glycosylation or S-glycosylation, respectively [85,86]. 

**Figure 10:**
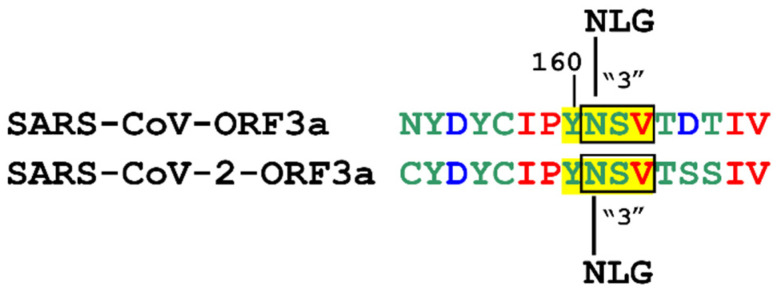
Prediction of atypical N-glycosylation PTMs (denoted by an open box) on the third SARS-CoV-ORF3a and SARS-CoV-2-ORF3 YXXΦ motif (in yellow). NLG: N-linked glycosylation.

It has been reported that SARS-CoV-2-ORF3a possesses three atypical NXV motifs, namely the NFV (119–121 aa), the NSV (161–164 aa), which overlaps the third YXXΦ motif, and the NPV (257–259 aa) [87]. By analogy, we expected that SARS-CoV-ORF3a would also possess the atypical NSV motif in the same position (161–164 aa). Indeed, results from the GlycoEP prediction server [60] showed that the Asn161 residues of the SARS-CoV-ORF3a and SARS-CoV-2-ORF3a proteins may be potentially N-glycosylated with approximately the same scores (Figure 10). Thus, this finding suggests a conserved PTM between the two viral proteins.

#### 3.3.8. S-Glutathionylation

**Figure 11:**
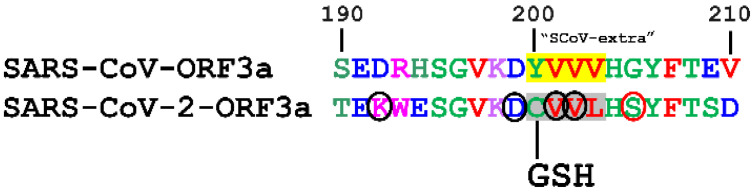
Prediction of a putative S-glutathionylation on SARS-CoV-2-ORF3a CVVL tetrapeptide (in grey). The residues indicated in black circles promote Cys glutathionylation, while the residues in red circles may inhibit it. The corresponding YVVV motif of SARS-CoV-ORF3a protein is shown in yellow. GSH: Glutathionylation.

S-glutathionylation is a reversible addition of a glutathione donor (GSSG) to the thiol side group (SH) of Cys residues. S-glutathionylation prediction was carried out using the DeepGSH server [61] and demonstrated increased probability for S-glutathionylation of the Cys200 residue of SARS-CoV-2-ORF3a (Figure 11). Near Cys200, residues Asp (at −1 position), Val (at +1 and +2 positions) and the distal Lys (at −8 position). were predicted to facilitate the putative occurrence of S-glutathionylation, as suggested elsewhere [88]. Conversely, the Ser (at +5 position) could hinder this PTM. Table S11 depicts the relevant prediction data for S-glutathionylation.

#### 3.3.9. N-Myristoylation

**Figure 12:**
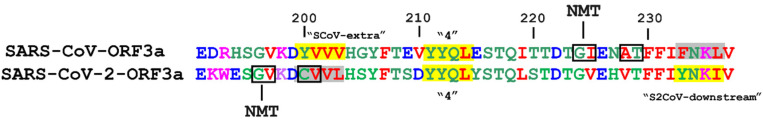
Location of putative N-myristoylation sites in SARS-CoV-ORF3a and SARS-CoV-2-ORF3a proteins. Residues noted with boxes belong to the myristoylation motif and partially overlap or are found adjacently to YXXΦ motifs (in yellow) and YXXΦ-like tetrapeptides (in grey). NMT: N-Myristoylation.

N-Myristoylation is a PTM that usually occurs at exposed glycine residues, when a myristoyl group derived from myristic acid is covalently linked via an amide bond to the glycine residue, a reaction catalysed by an N-myristoyltransferase [87]. The suggested N-myristoylation consensus sequence model (ELMpattern PS00008) is described as G–{EDRKHPFYW}–XX–[STAGCN]– [23]. PROSITE analyses [62] revealed that an N- myristoylation site in SARS-CoV-ORF3a may occur at a GIenAT peptide at position 224–229, in proximity to the SCoV-extra and the fourth YXXΦ motifs. In SARS-CoV-2-ORF3a, the N-myristoylation site GVkdCV is predicted at position 196–201, where the Cys200 is absolutely essential for its formation and neighbouring Val197 and Val201 residues may enhance the PTM (Figure 12). In silico mutagenesis of C200 to Y200 was performed to mimic the corresponding amino acid sequence of SARS- CoV-ORF3a and resulted in loss of the N-myristoylation site, thereby confirming the importance of Cys200. Similarly, in silico mutagenesis in SARS-CoV-ORF3a of Ala228Val and Ile225Val abrogates the N-myristoylation site. 

#### 3.3.10. Sulfation

**Figure 13:**
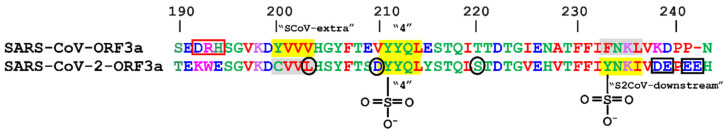
Prediction of putative sulfation PTMs on SARS-CoV-2-ORF3a YXXΦ motifs (in yellow). The residues indicated in black circles or boxes promote sulfation, while the residues in the open red box could inhibit it. YXXΦ–like tetrapeptides of both proteins are depicted in grey.

Tyrosine sulfation constitutes a critical PTM, where a sulfate group is added to a Tyr residue of a protein. This PTM has been suggested to expose Tyr residues on the surface of the protein. [89]. In silico analyses of Tyr sulfation with the GPS-TSP prediction server [63], demonstrated that SARS-CoV-2-ORF3a possesses two putative tyrosine sulfation sites, at position 211, located in the fourth YXXΦ motif (211–214 aa), and at position 233, located in the S2CoV-downstream YXXΦ motif (233–236 aa). As far as Tyr211 is concerned, sulfation could be facilitated by the presence of the acidic residue Asp (at −1 position), the Leu (at −8 position) and the Ser (at +9 position), all of which promote tyrosine sulfation at varying degrees [90]. As expected, the adjacent Tyr212 residue demonstrated a very low sulfation probability (score < 1, see Table S12). Equally, the Tyr211 of SARS-CoV-ORF3a protein also exhibited a very low sulfation probability (score < 1, see Table S12), despite the existence of the acidic Glu209 residue at −2 position, presumably because of the existence of Val210 residue at −1 position, which confers an overall restrictive effect on the ability of Tyr211 to be sulfated. Furthermore, the Tyr233 residue of SARS-CoV-2-ORF3a could become sulfated. This putative PTM was predicted to be enhanced by the presence of the dipeptide Asp-Glu (at +5 and +6 positions) and to a lesser extent by the Glu-Glu residues (at +8 and +9 positions, respectively), which are unique and characteristic to the SARS-CoV-2 viral protein (Figure 13).

Surprisingly, the Tyr200 residue of the SARS-CoV-ORF3a protein, located in the SCoV-extra YXXΦ motif, was not predicted to be sulfated, despite the presence of Asp199 at position −1 (score <1 see Table S12), possibly because of the existence of the DRH (Asp-Arg-His) tripeptide located at position 192–194, which was predicted to obstruct Tyr200 sulfation.

### 3.4. 3D Structures of the ORF3a Viral Proteins

**Figure 14:**
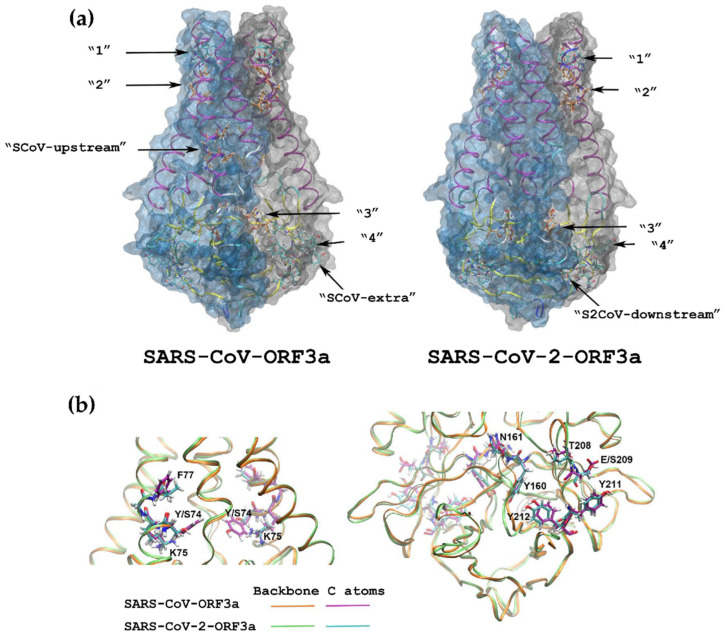
(**a**) Three-dimensional structures of SARS-CoV-ORF3a (structure on the left) and SARS-CoV-2-ORF3a (structure on the right) dimers. Residues of the YXXΦ motifs are shown as sticks and indicated by arrows. In the case of the fourth conserved motif, the SCoV-extra and the S2CoV-downstream motifs, the arrows point at the Tyr residue of the tetrapeptides. The carbon atoms of the motifs where Tyr is located inside the dimer’s interface are coloured in purple, while the ones facing the exterior are coloured in cyan. (**b**) Superimposition of the SARS-CoV-ORF3a dimer with that of the SARS-CoV-2-ORF3a one, focusing on the areas of the alternative conduction pathway (see reference [50] for a definition of the pathway). The figures were prepared with VMD 1.9.3.

A previous study by Kern and colleagues suggested that ORF3a dimerises and forms an ion channel permeable to calcium [50]. Based on this work, we mapped the positions of the YXXΦ motifs of SARS-CoV ORF3a and SARS-CoV-2 ORF3a in a 3D environment, using the Modeller V9.22 software [51]. Figure 14a depicts these 3D structures, where it is possible to discern all putative YXXΦ motifs, indicated by arrows. Figure 14b shows an enlarged view of the key amino acids of the second motif (left panel) and the third and fourth motifs (right panel), as an example of the way PTMs could affect the functionality of the channel, which will be discussed in the Discussion section. In this model, we have superimposed SARS-CoV-ORF3a sequence with that of SARS-CoV-2-ORF3a.

### 3.5. Immune-Related Functions of the SARS-CoV-ORF3a and SARS-CoV-2-ORF3a ΥΧΧΦ Motifs and ΥΧΧΦ-like Tetrapeptides

**Figure 15:**
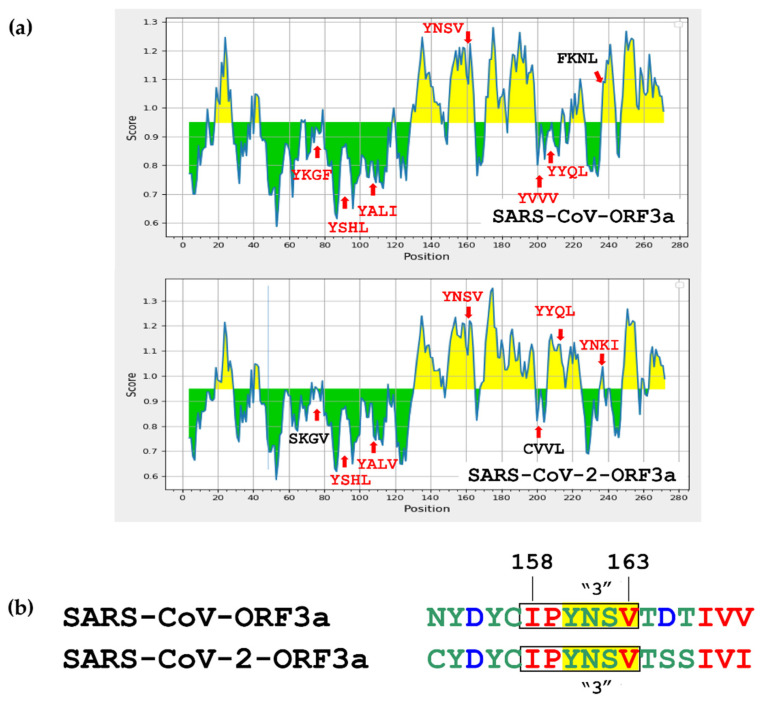
(**a**) Prediction of linear B-cell epitopes, on SARS-CoV-ORF3a and SARS-CoV-2-ORF3a ΥΧΧΦ motifs and ΥΧΧΦ-like tetrapeptides, using the Chou & Fasman method of Beta-turn prediction. Residues with scores above the threshold (0.95) are coloured in yellow and the ones below the threshold in green. (**b**) Prediction of a conserved ITIM motif overlapping the 3rd YXXΦ motif. Positions of YXXΦ motifs and YXXΦ-like tetrapeptides, are noted in red and black, respectively.

Recent studies have reported that the ORF3a of SARS-CoV and S2CoV may have antigenic properties [22,91]. We have investigated the possibility that the regions within and adjacent to the ΥΧΧΦ motifs and ΥΧΧΦ-like tetrapeptides of SARS-CoV ORF3a and SARS-CoV-2 ORF3a may display antibody epitopes, involved in B-cell-related immune responses. For this analysis, we used the Chou & Fasman Beta-turn prediction tool and data concerning the linear sequences of antigenic peptides together with their dedicated ID numbers from the IEDB Analysis Resource web server (see Figure 15a and Table 2 with scores and ID numbers). According to our prediction, the third YNSV motif (160–163 aa) belongs to an antigenic region in both ORF3a proteins. The two YNSV antigenic peptides (yellow area) of SARS-CoV-ORF3a and SARS-CoV-2-ORF3a had similar scores and were also detected as antigenic in in vitro B-cell assays [92,93]. Next, the fourth common YYQL motif (209–224 aa) was predicted to belong to a highly antigenic area in SARS-CoV-2-ORF3a but not in SARS-CoV-ORF3a (green area), verified in vitro elsewhere [94]. Similarly, the S2CoV-downstream YNKI motif (233–236 aa) was predicted to show antigenic properties, validated by Schwarz and colleagues [93]. Notably, substitution of the downstream YXXΦ-like (FKNL) tetrapeptide of SARS-CoV with a typical YXXΦ motif (YNKI) in SARS-CoV-2 resulted in reduced in antigenicity for the emerging virus. No other differences were observed for the rest of the YXXΦ motifs and ΥΧΧΦ-like tetrapeptides between the two viral proteins.

For the prediction of conformational antigenic epitopes, we used the Epitope3D web server [65]. The tool was loaded with the cryo-EM structure of the SARS-CoV-2-ORF3a protein (PDB ID: 7KJR; chains A and B), [50] and the model approximation of SARS-CoV-ORF3a we prepared for our structural analysis (Figure 14a), since there is no published crystal structure for the latter. This analysis provided antigenic amino acids located in the SKGV YXXΦ-like tetrapeptide of SARS-CoV-2, the YSHL motif in both viral proteins, the YNSV motif of SARS-CoV-2 and the FKNL YXXΦ-like tetrapeptide of SARS-CoV. File S1 contains all antigenic residues for both ORF3a proteins.

Finally, we investigated the possibility that one of the YXXΦ motifs or YXXΦ-like tetrapetides on the viral ORF3a proteins could be part of an ITAM/ITIM motif, as it has recently been suggested that such motifs found on viral proteins are involved in the regulation of host immune evasion strategies mounted by viruses [95]. Interestingly, ELM protein analyses showed that YNSV tetrapeptide participates in the ITIM Tyr-based Immunoreceptor motif IPYNSV, which lies at position 158–163 aa (Figure 15b). This important motif is conserved in both viral proteins.

## 4. Discussion

The present study attempts to investigate putative biochemical features such as PTMs, which centred specific functions to the ORF3a accessory protein of the emerging coronaviruses SARS-CoV and SARS-CoV-2. Our approach focuses on the presence of the YXXΦ motifs, which are widely used by viruses for endocytosis, egress, structural integrity of the viral capsid and the completion of other parts of the viral life cycle [32,35]. Firstly, we mapped the predicted motifs on existing structural elements of the two proteins, and then we carried out computational predictions of the most commonly encountered PTMs occurring on their amino acid sequences and highlighted crucial differences between the two viral proteins.

Despite being small and overlooked, the OF3a proteins of both SARS viruses appear to uphold some key duties in the viral life cycle, pathogenesis and immune regulation of the host, beautifully reviewed by Zhang and colleagues [96]. Interestingly, the YXXΦ motif at 160–163 aa seems to be conserved in both proteins. If destroyed by mutagenesis, the protein’s membrane association is abolished and its function as an apoptosis inducer is severely downplayed [27]. To our knowledge, no other YXXΦ motifs have been experimentally verified to date, however, prediction analyses showed that there could be more active motifs in ORF3a proteins (Figure 2a). Furthermore, differences in the amino acid sequence between the two viruses suggest that single- or double-aa substitutions create YXXΦ-like tetrapeptides that may be capable of being subjected to certain PTMs. Of course, the consensus pattern of the YXXΦ motif is not the absolute reason for functionality. Other factors, such as the presence of di-leucine residues, the surrounding amino acids or the presence of intrinsically disordered regions, as the ones identified near or between some of the putative YXXΦ motifs on ORF3a proteins (Figure 3b) have been proposed to influence the putative function of various motifs [97].

Phosphorylation plays a key role in cellular signalling, enzymatic activity and several other cellular processes, including regulation of the immune response [74]. Phosphorylation of viral proteins can have a significant impact on infection by both RNA and DNA viruses, their replication and cytotoxicity in a host cell. In fact, phosphorylation may regulate interactions between viral proteins and the viral genome throughout the well-orchestrated viral life cycle, viral protein stability, as well as virus–host interactions [98,99]. This is true for the SARS-CoV-2 virus also, where many phosphorylation sites have already been predicted for several SARS-CoV-2 proteins, including ORF3 proteins [38,100]. Some of the predicted YXXΦ motifs may have the potential to be phosphorylated, surely under specific circumstances, and many predicted sites are conserved between the two ORF3a viral proteins. It has been previously shown that phosphorylation of the Tyr residue on a YXXΦ sorting motif inhibits endocytosis of the YXXΦ-bearing protein, because the addition of the phosphate group stereochemically blocks binding of the adaptor protein AP-2M1 on the motif [101,102]. It has also been recently suggested that phosphorylation of a Ser residue within a YXXΦ motif might affect protein sorting but not endocytosis itself, as in the case of the endocytic pathway transporter sortilin [103]. Thus, whether phosphorylation of a Tyr or Ser residue, such as the ones found in some of the YXXΦ motifs or YXXΦ-like tetrapeptides of SARS-CoV-ORF3a and SARS-CoV-2-ORF3a (Figure 4), induces such a change in ORF3a localisation and subsequent function should be experimentally assessed for each individual functional motif.

Protein ubiquitination coordinates the cellular localisation of proteins by modulating protein-protein interactions and activation status. It also regulates a variety of cellular processes, including cell division and mitosis, signal transduction, endocytosis and membrane trafficking associated with several viruses [104]. As an innate mechanism to counter viral infection, the host performs ubiquitination of viral proteins and targets them for proteasomal degradation, limiting the viral spread or affecting the replicon complex [105]. Alternatively, viruses themselves may use the host ubiquitination system in order to control replication, entry tropism, spread and viral protein stability with flaviviruses, such as the Zika virus, or the HPV DNA viruses, being typical examples [106]. Finally, increasing evidence suggests that many viruses have evolved evasion strategies that specifically use or disable ubiquitin-dependent responses through expression of viral ubiquitin-like molecules, ubiquitin ligases, and deubiquitinases [107]. We observed that the predicted SCoV-upstream YXXΦ motif, partially located within the first TM region of the protein, turns into a YXXΦ-like tetrapeptide capable of ubiquitination in the SARS-CoV-2-ORF3a, with the possibility of conferring a ubiquitin-related function to ORF3a (Figure 5).

Succinylation was predicted to occur only in SARS-CoV-ORF3a and to be strongly inhibited by neighbouring amino acids in the corresponding SARS-CoV-2 protein (Figure 6). This PTM is largely understudied, but it is known to alter protein structure and induce negative charge to modified proteins. It has been shown to respond to metabolic changes, linked to fatty acid metabolism and implicated in cardiovascular and Alzheimer’s disease [108,109]. Because the succinyl group is considered to be much larger than the phosphoryl group added during protein phosphorylation, if such a PTM occurs on an YXXΦ motif, it is likely to inhibit its function as a sorting motif. Hence, one could hypothesise that ongoing evolution of the SARS virus would have removed the potential for succinylation from the YXXΦ motif. So far, there have been no reports about ORF3a succinylation, however, the non-structural protein SARS-CoV-2 nsp14, which is crucial for viral replication, has been proposed to positively interact with SIRT5 [110], a factor well-known for activating succinylated protein substrates through desuccinylation [108].

The role of methylation and acetylation in epigenetic regulation of histones, gene transcription and other major cellular functions is well established [78,79,111,112]. Recently, Ahmed and colleagues reported that at least eight IAV proteins could be methylated and acetylated by relevant host enzymes in specific Lys residues. The modified proteins held crucial roles in viral entry, replication, virion assembly and host immune evasion [113]. Furthermore, it has been shown that the N protein of both SARS-CoV-2 and SARS-CoV is acetylated in vitro by host PCAF and GCN5 acetylases, however, it remains unknown how this affects protein functionality [114]. Our data predicted multiple methylation events in the YXXΦ motifs of both ORF3a proteins (Figure 7) and a single acetylation modification in an YXXΦ-like tetrapeptide of the SARS-CoV-2-ORF3a (Figure 8). The putative role of these PTMs remains elusive and warrants further investigation.

Tyrosine nitration induces changes related to structure and protein functions, including sensitivity to proteolysis, alterations in phosphorylation cascades [115], induction of immunological responses by the generation of antibodies against nitrated proteins [116] and attenuation of innate immunity mechanisms. Nitrated Tyr residues have been reported in Adenoviruses type 2 viral proteins, possibly portraying a metabolically stressed host cell during viral assembly [117]. Another report on the zoonotic lymphocytic choriomeningitis virus (LCMV) has offered proof of concept related to the use of nitrated Tyr residues of the LCMV glycoprotein gp33, as recognition epitopes by T-cells under inflammatory conditions mediated by viral infection [116]. SARS-CoV-2 has been suggested to cause NO-related stress in vivo [118], however, whether any of the viral proteins are regulated through nitration remains unknown. Notably, it has been suggested that nitration of S protein has deleterious effects on viral entry and it actually inactivates the virus due to extensive nitrotyrosine formation [119]. This study successfully demonstrates the feasibility of using induced PTMs as therapeutic means or environmental anti-infectious agents. We have predicted two putative nitration events, a Tyr nitration in SARS-CoV-ORF3a, which is then abolished in SARS-CoV-2 and an S-nitrosylation event on the Cys200 residue of SARS-CoV-2-ORF3a (Figure 9). The latter appears exposed to the intracellular cytosolic side and has been shown to be conserved in SARS-CoV-2 from pangolin or bats [12]. This implies that the Y200C mutation was not random, but it was conserved during the cross-species jump of SARS-CoV-2 and could play yet unidentified roles in the interaction of the protein with cytosolic host partners. Thus, it is possible that nitration or any other putative PTM on this residue could have an important effect for the survival of the virus.

N-glycosylation is an important PTM that does not cause significant changes in the structure of the protein but, rather, regulates function. About half of the collective proteome in nature is predicted to be glycosylated [120]. N-glycosylation is implicated in an increasing number of biological procedures, such as cell adhesion, cell-cell communication, protein folding and signal transduction as reviewed in [71]. Many viruses possess glycosylated proteins involved in all the above cellular processes and participating in the completion of crucial steps of the viral life cycle, such as the involvement of hepatitis B virus (HBV) surface proteins in proper virion egress [121]. There is a highly conserved atypical N-glycosylation signal on the 160–163 aa YXXΦ motif of both SARS viruses (Figure 10). The one on SARS-CoV-2-ORF3a has already been reported [122] and we can only assume that conservation of this PTM on the ORF3a internalisation motif between the two viruses may play a yet unidentified functional role for this protein.

S-glutathionylation primarily mirrors the oxidative stress status of the cell. It has been shown that this PTM plays key roles in cell signalling, TNF-α-dependent apoptosis and antiviral host responses [123]. Indeed, a recent study demonstrated that HCV NS5B RNA-dependent RNA polymerase enzymatic activity is gravely down-regulated by S-glutathionylation. This effect has also been observed in other viruses, for instance members of the *Flaviviridae* family, the Chikungunya virus and HIV [124]. The putative S-GSH site created on the YXXΦ-like tetrapeptide of SARS-CoV-2-ORF3a protein with the abolishment of the corresponding SCoV-extra motif (Figure 11) could initiate new interactions with host proteins or be a means for host-mediated control of the virus.

N-myristoylation is important for proteins involved in diverse biological processes such as signal transduction, cellular localisation, oncogenesis, innate immunity and immune surveillance mechanisms and plays an important role in protein stability and localisation of proteins to membranes [87,125]. Several studies report that myristoylated viral proteins are crucial for replication, assembly, budding and viral entry. Examples include the Gag protein of HIV [126] and the Vp4 capsid protein of picornaviruses [127]. HBV receptor recognition occurs over an N-terminal QLDPAF sequence in the vicinity of a myristoylation motif on the pre-S1 domain of HBV L protein. This is essential for entry and infectivity not only for HBV but also for the satellite hepatitis D virus (HDV), which shares part of the HBV life cycle [128,129]. As for SARS-CoV-2, a recent study suggested that the structural proteins S and M were predicted myristoylated in silico, thereby signalling a cascade of molecular events relevant to completion of the viral life cycle but possibly initiation of inflammatory pathways too [130]. We predicted a single N-myristoylation PTM on both ORF3a proteins located close to, but on opposite sides of, the putative fourth YXXΦ motif (Figure 12). Conservation of the motif may underline its implication into the host immune response and should be investigated further.

Protein sulfation drastically changes both the molecular mass and charge of a protein, and at the same time prevents it from degradation through peroxidation or proteolysis of the sulfhydryl group [131]. Sulfated Tyr residues are involved in many biological functions, including host–pathogen interactions and ligand binding [89]. Virus entry is also facilitated, as suggested for the HIV and CMV viruses, albeit in different ways. Specifically, sulfation of the CMV protein UL22A enhances its binding to the chemokine RANTES, thereby inhibiting it from binding to chemokine receptors and initiating host antiviral responses [132]. In contrast, HIV induces sulfation of its cellular co-receptor CCR5 to achieve cell entry [133]. Our study predicts two sulphated Tyr residues within the SARS-CoV-2-ORF3a fourth and the S2CoV-downstream YXXΦ motifs only (Figure 13). Given that sulfation increases the molecular weight of a protein similarly to phosphorylation and also exposes the modified residues to the cell surface [89,117], one would expect that, upon sulfation, the ORF3a protein could engage in new, still undiscovered interactions with extracellular host proteins. 

In order to probe the role of putative PTMs on the functionality of the predicted YXXΦ motifs further, we generated three-dimensional models of the two ORF3a proteins to visualise the position the predicted YXXΦ motifs on the protein body (Figure 14a). The dimeric fold of the protein forms a polar cytoplasmic cavity conducting cations halfway across the membrane with the opening of the channel to the outer half being sealed through hydrophobic interactions between the transmembrane α-helices [50]. Two of the conserved YXXΦ motifs (first and second motif) are located in the vicinity of this hydrophobic seal in the three-dimensional structures of both SARS-CoV and SARS-CoV-2 ORF3a proteins. Therefore, post-translational modifications in that area may induce conformational changes, leading to the breakage the hydrophobic seal, thereby opening the channel to the exterior of the membrane. In such a scenario, the second and third conserved motifs along with the SCoV-upstream motif would affect cation transfer as their hydrophilic residues are located inside the pore. According to Kern and colleagues [50], an alternate conduction pathway could be formed in membrane-facing hydrophilic cavities at the interface of transmembrane helices 2 and 3. In that case, the conserved Lys75 located in the upper part of this pathway (Figure 14b; left panel), and the Lys160-Asn161 of the third YXXΦ motif positioned in the lower portion of the cavity (Figure 14b; right panel), could very well assist with cation conducting, since according to our results both positions are predicted to be post-translationally modified. Specifically, Lys75 may be prone to various modifications, such as succinylation, methylation, acetylation and ubiquitination, while the Lys160-Asn161-containing motif could be subjected to phosphorylation or N-glycosylation. Of note, in our three-dimensional models the side chain of Lys75 is positioned away from Ser74 in SARS-CoV-2-ORF3a, while in SARS-CoV-ORF3a Tyr74 is interacting with the lysine’s amino group (Figure 14b; left panel). Nonetheless, as Ser74 might potentially be phosphorylated, this interaction could be restored in the SARS-CoV-2-ORF3a. Lastly, the side chains of amino acids predicted to form a phosphorylated patch in SARS-CoV-2-ORF3a (i.e., Thr208, Ser209, Tyr211 and, Tyr212) are located in the solvent accessible surface of the protein except that of threonine at position 208 (Figure 14b; right panel), therefore they may be more accessible to phosphorylation by host kinases. Thus, one could hypothesise that such PTMs in the putative YXXΦ motifs might be beneficial for the viroporin-related function of ORF3a in both viruses, or they can lead to a variety of ORF3a interactions with host proteins.

Visualisation of the YXXΦ motifs and studying the topological features of a protein can certainly help the reader imagine the putative role of a PTM on protein function. Still, without experimental verification, the usefulness of these predictions may be easily questioned, as the algorithms and servers used to acquire them could have low predictive power. Unfortunately, validation studies on the input of PTMs on YXXΦ functionality as sorting motifs or change of function are still scarce, especially when it comes to viral proteins. Interestingly, the ones that exist may train the prediction servers, so that they achieve more reliable data. One such proof-of-concept example is the study by Fruehling and Longnecker, who investigated how functionality of the LMP-2A protein of Epstein-Barr Virus (EBV) was altered following Tyr phosphorylation of its YXXΦ motif [134]. The data were used by the group of Schwartz and Church who created a viral PTM database that accurately predicts phosphorylation sites in human viruses [135] (p. 3).

Finally, we looked for immune-related functions that could be associated with the YXXΦ motifs and YXXΦ-like tetrapeptides predicted in SARS-CoV-ORF3a and SARS-CoV-2-ORF3a proteins. It has been recently reported that patients suffering from COVID-19 disease develop antibodies against ORF3a protein. About 7% of recovered patients mounted anti-ORF3a antibodies [136]. Another study reported significant recognition of ORF3a peptides by CD4+ T-cells in patients suffering with the mild form of the disease [137]. Given, that ORF3a has exhibited antigenic properties in vivo, we speculated that some of the predicted YXXΦ motifs and YXXΦ-like tetrapeptides in both viruses could be responsible for some of these properties oriented against B cell responses. Indeed, analysis with the Chou & Fasman method showed that each viral protein retains two motifs that could display antigenic properties (Figure 15a). Interestingly, both ORF3a proteins revealed a higher antigenicity at the second half of their sequence (130–270 aa) as a result of the presence of several β-sheets. Βeta-turns that compose these β-sheets can change the direction of the main peptidic chain and attribute to the protein properties such as surface accessibility, hydrophilicity and flexibility, which are related to high antigenicity [138].Several of these linear epitopes have been experimentally validated [92,93,94] and this suggests the functional importance of YXXΦ motifs in virus-host immune responses. Importantly, there is an overlap between linear and conformational epitope prediction concerning the YNSV motif (160–163 aa) for both proteins and the FKNL tetrapeptide (233–236 aa) in SARS-CoV. Of course, this variability may mirror the fundamental differences between linear and discontinuous B-cell epitopes [139]. Notably, the YXXΦ motif in the 160–163 aa position, was predicted to be part of an ITIM motif and its role in SARS-CoV-2 pathogenesis through engagement of surface receptors and accompanying signalling is part of ongoing studies in the laboratory.

In conclusion, although SARS-CoV-ORF3a and SARS-CoV-2-ORF3a proteins are approximately the same size, they show extensive differences in their N-terminus, as well as significant and variable point mutations throughout the length of their amino acid sequences, especially within the predicted YXXΦ motifs. The predicted PTMs and structural features we discussed in this paper could attribute diverse functions to the ORF3a proteins, firstly by destruction of the YXXΦ sorting motifs, bearing implications for protein localisation, and secondly by mediating interactions with host proteins involved in immune signalling and other cellular responses. Although it is difficult to draw conclusions about the biological functions of a protein from bioinformatically predicted structures, we hope that the results of our work will boost in vitro experimentation on this fascinating biomolecule, ORF3a that will ultimately assign new biological roles to it. Furthermore, we envision that the targeting of the YXXΦ motifs with pharmacological reagents that will induce PTMs with suitable deleterious effects to the structural integrity and the immunological properties of ORF3a, could assist with the rational design of anti-SARS-CoV-2 treatments.

## Figures and Tables

**Table 1:** Presentation of the canonical ΥΧΧΦ [M/L/I/V/F] motifs within the SARS-CoV-ORF3a and SARS-CoV-2-ORF3a protein sequences.

SARS-CoV-ORF3a			SARS-CoV-2-ORF3a		
Canonical YXXΦ Motifs					
Peptide	Position	Motif	Peptide	Position	Motif
YKGF *	74–77	SCoV-upstream	-	-	
YSHL *	91–94	1st	YSHL	91–94	1st
YALI *	109–112	2nd	YALV	109–112	2nd
YNSV	160–163	3rd	YNSV	160–163	3rd
YVVV	200–203	SCoV-extra	-	-	
YYQL	211–214	4th	YYQL	211–214	4th
-	-		YNKI	233–236	S2CoV-downstream

* ΥΧΧΦ motifs with a low score according to ELM protein analysis.

**Table 2:** List of the peptide sequences of the predicted linear B-cell epitopes accompanied by their IEDB ID number, sequence position and average score according to the Chou & Fasman prediction analyses.

SARS-CoV-ORF3a				
ID	Start	End	Sequence	Average Score
1446523	154	165	HDYCIP**YNSV**TD	1.155
**SARS-CoV-2-ORF3a**				
**ID**	**Start**	**End**	**Sequence**	**Average Score**
1540003	155	169	DYCIP**YNSV**TSSIVI	1.157
1692668	209	224	SD**YYQL**YSTQLSTDTG	1.081
1543496	233	247	**YNKI**VDEPEEHVQIH	1.037

## Data Availability

Not applicable.

## Supplementary Materials

The following supporting information can be downloaded at: https://www.mdpi.com/article/10.3390/biom12081052/s1, Table S1: Prediction of the non-canonical YXXXΦ[M/L/I/V/F] motifs, the reverse [M/L/I/V/F] ΦXXY motifs and the di-leucine motifs within the SARS-CoV-ORF3a and SARS-CoV-2-ORF3a; Table S2: Predicted TM helices topology within the SARS-CoV-ORF3a and SARS-CoV-2-ORF3a; Table S3: Predicted α-helices, β-sheets and coil segments within the SARS-CoV-ORF3a and SARS-CoV-2-ORF3a; Table S4: Predicted phopshorylated Ser, Tyr and Thr residues (in red) located within the YXΧΦ motifs, the YXΧΦ-like tetrapeptides or adjacently to them, for SARS-CoV-ORF3a and SARS-CoV-2-ORF3a. The kinase catalysing each phoshorylation is indicated; Table S5: Predicted ubiquitinated Lys (in red) located within the YYΧΦ-like tetrapeptides and proximal regions of SARS-CoV-ORF3a and SARS-CoV-2-ORF3a; Table S6: In silico mutagenesis analysis for the adjacent residues (in red) to the Lys75 of the 1st YXΧΦ-like tetrapeptide and ubiquitynilation prediction for these mutated sequences in SARS-CoV-2-ORF3a; Table S7: Predicted methylated Lys residues (in red) of SARS-CoV-ORF3a and SARS-CoV-2-ORF3a; Table S8: Predicted acetylated Lys residues (in red) of SARS-CoV-ORF3a and SARS-CoV-2-ORF3a; Table S9: Predicted nitrated Tyr residues of SARS-CoV-ORF3a and SARS-CoV-2-ORF3a; Table S10: Predicted S-nitrosylated cysteines (in red) of SARS-CoV-ORF3a and SARS-CoV-2-ORF3a; Table S11. Predicted S-glutathionylated Cys residues (in red) of SARS-CoV-ORF3a and SARS-CoV-2-ORF3a; Table S12: Predicted sulfated Tyr residues (in red) of SARS-CoV-ORF3a and SARS-CoV-2-ORF3a. File S1: Predicted Linear and Conformational Antigenicity Data.
