# Supplementary material for: Targeting the YXXΦ Motifs of the SARS Coronaviruses 1 and 2 ORF3a Peptides by In Silico Analysis to Predict Novel Virus—Host Interactions
Source: Biomolecules. 2022 Jul 29;12(8):1052. doi: 10.3390/biom12081052 (PMC9405953; doi:10.3390/biom12081052)
Supplement: Supplementary file 1 [file biomolecules-12-01052-s001.zip › Supplementary Tables Kakkanas et al 2022.pdf]

Table S1. Presentation of the non-canonical YXXXΦ [M/L/I/V/F] motifs, the reverse [M/L/I/V/F] ΦXXY motifs and the di-leucine motifs within the SARS-CoV-ORF3a and SARS-CoV-2-ORF3a.

SARS-CoV-ORF3a		SARS-CoV-2-ORF3a	
**Non-canonical YXXXΦ motifs**			
**peptide**	**position**	**peptide**	**position**
YLYAI	107 - 111	YLYAV	107 - 111
YDYCI	154 - 158	YDYCI	154 - 158
YFTEV	206 - 210	-	-
-		YSTQL	215 - 219
**Reverse ΦXXY motifs**			
**peptide**	**position**	**peptide**	**position**
LALY	71 74	-	-
VTIY	88 - 91	VTVY	88 - 91
LYLY	106 - 109	LYLY	106 - 109
IGGY	186 -189	IGGY	186 -189
VKDY	197 – 200	-	-
VHGY	203 - 206	LHSY	203 - 206
-	-	FFIY	230 - 233
**Di-leucine motifs**			
**peptide**	**position**	**peptide**	**position**
SKNPLL	135-140	SKNPLL	135-140

Table S2. Predicted transmembrane (TM) helices topology within the SARS-CoV-ORF3a and SARS-CoV-2-ORF3a.

SARS-COV-ORF3a		SARS-COV-2-ORF3a	
**topology**	**residues**	**location**	**residues**
outside	1 - 42	outside	1 - 34
1st TMhelix	43 - 64	1st TMhelix	35 - 56
inside	65 - 75	inside	57 - 76
2nd TMhelix	76 - 99	2nd TMhelix	77 - 101
outside	100 - 104	outside	102 - 106
3rd TMhelix	105 - 125	3rd TMhelix	107- 129
inside	126 - 274	inside	130 - 275

Table S3. Predicted α-helices, β-sheets and coil segments within the SARS-CoV-ORF3a and SARS-CoV-2-ORF3a.

**SARS-CoV-ORF3a**		**SARS-CoV-2-ORF3a**	
**structure**	**position**	**structure**	**position**
1st coil	1-3	1st coil	1-3
1st α-helix	4-5	-	-
1st β-sheet	6-10	1st β-sheet	4-15
2nd coil	11-17	2nd coil	16-28
2nd β-sheet	18-20	-	-
3^{d} coil	21-28	-	-
3rd β-sheet	29-35	2nd β-sheet	29-38
4th β-sheet	38	-	-
4th coil	39-43	3rd coil	39-43
2nd α-helix	44-58	1st α-helix	44-57
5th coil	59	4th coil	58-61
3rd α-helix	60-61	-	-
5th β-sheet	62-65	2nd β-sheet	62-65
5th coil	66-67	-	-
4th α-helix	68-75	2nd α-helix	66-74
6th coil	76	5th coil	75-76
5th α-helix	77-98	3rd α-helix	77-99
7th coil	99-101	6th coil	101-103
6th α-helix	102-130	4th α-helix	104-132
6th β-sheet	131-132	-	-
8th coil	133-139	6th coil	133-139
7th β-sheet	140-142	3rd β-sheet	140-142
9th coil	143-144	7th coil	143-144
8th β-sheet	145-150	4th β-sheet	145-150
9th β-sheet	156-158	5th β-sheet	156-158
10th coil	159-164	8th coil	159-163
10th β-sheet	165-170	6th β-sheet	164-170
11th coil	171-199	9th coil	171-190
-	-	5th α-helix	191 - 194
-	-	10th coil	195-199
10th β-sheet	200-205	7th β-sheet	200-204
12th coil	206	11th coil	205-211
11th β-sheet	207-215	8th β-sheet	212-219
12th coil	216	-	-
11th β-sheet	217-220	-	-
13th coil	221-228	12th coil	220-225
12th β-sheet	229 - 233	9th β-sheet	226 - 234
7th α-helix	234 - 236	-	-
14th coil	237-243	12th coil	235-243
13th β-sheet	244 - 248	10th β-sheet	244 - 249
15th coil	249-274	13th coil	250-275

Table S4. Predicted phopshorylated Ser, Tyr and Thr residues (in red) located within the YXXΦ motifs, the YXXΦ-like tetrapeptides or adjacently to them, for SARS-CoV-ORF3a and SARS-CoV-2-ORF3a. The kinase catalysing each phoshorylation is indicated.

SARS-CoV-ORF3a				
**position**	**YXXΦ motif**	**sequence**	**score**	**kinase**
-	-	-	-	-
S92	1st	VTIYSHLLL	0.512	PKA
Y160	3rd	YCIPYNSVT	0.525	unspecified
S162	3rd	IPYNSVTDT	0.971	unspecified
Y212	4th	TEVYYQLES	0.532	SRC
T217	-	QLESTQITT	0.518	DNAPK
**SARS-CoV-2-ORF3a**				
**position**	**YXXΦ motif**	**sequence**	**score**	**kinase**
S74	upstream	QLALSKGVH	0.706/<br>0.535	PKC/<br>cdc2
S92	1st	VTVYSHLLL	0.503	cdc2
Y160	3rd	YCIPYNSVT	0.525	unspecified
T208	-	HSYFTSDYY	0.774	PKC
S209	-	SYFTSDYYQ	0.516/<br>0.502	CKII/<br>PKA
Y211	4th	FTSDYYQLY	0.698/<br>0.518	unspecified<br>NSR
Y212	4th	TEVYYQLES	0.512	SRC

Table S5. Predicted ubiquitinated Lys (in red) located within the YYXΦ-like tetrapeptides and proximal regions of SARS-CoV-ORF3a and SARS-CoV-2-ORF3a.

SARS-CoV-ORF3a			
**position**	**YXXΦ-motif**	**sequence**	**score**
K19	-	SITAQPVKIDNASPA	0.64
K61	-	AVFQSATKIIALNKR	1.83
K132	-	MRCWLCWKCKSKNPL	1.74
K181	-	GISTPKLKEDYQIGG	0.52
K235	downstream-like	ATFFIFNKLVKDPPN	0.31
**SARS-CoV-2-ORF3a**			
**position**	**YXXΦ-motif**	**sequence**	**score**
K16	-	TIGTVTLKQGEIKDA	0.63
K21	-	TLKQGEIKDATPSDF	1.13
K61	-	AVFQSASKIITLKKR	2.46
K66	-	ASKIITLKKRWQLAL	1.42
K67	-	SKIITLKKRWQLALS	0.46
K75	upstream-like	RWQLALSKGVHFVCN	0.98
K132	-	MRLWLCWKCRSKNPL	0.91

Threshold was set to 0.3

Table S6. *In silico* mutagenesis analysis for the adjacent residues (in red) to the Lys75 of the 1^{st}YXXΦ-like tetrapeptide and ubiquitination prediction for these mutated sequences in SARS-CoV-2-ORF3a.

	**SARS-CoV-2-ORF3a**	
	**amino acids**	**score**
**consensus**	SKGVHF	0.98
**mutated**	YKGVHF	<0.3
**mutated**	SKGFHF	0.48
**mutated**	SKGVQF	0.74

Threshold was set to 0.3

Table S7. Predicted methylated Lys residues (in red) of SARS-CoV-ORF3a and SARS-CoV-2-ORF3a.

**SARS-CoV-ORF3a**		**SARS-CoV-2-ORF3a**	
**position**	**YXXΦ-motif**	**position**	**YXXΦ-motif**
-	-	K21	-
-	-	K66	-
K75	upstream	-	
-	-	K136	-
-	-	K192	-
K235	-	K235	downstream
K238	-	-	-

Table S8. Predicted acetylated Lys residues (in red) of SARS-CoV-ORF3a and SARS-CoV-2-ORF3a.

**SARS-CoV-ORF3a**			
**position**	**YXXΦ motif**	**sequence**	**score**
K61	-	VFQSATKIIALNK	1.32
K132	-	RCWLCWKCKSKNP	1.67
K134	-	WLCWKCKSKNPLL	0.64
K198	-	DRHSGVKDYVVVH	0.27
**SARS-CoV-2-ORF3a**			
**position**	**YXXΦ motif**	**sequence**	**score**
K16	-	IGTVTLKQGEIKD	0.92
K21	-	LKQGEIKDATPSD	1.46
K61	-	VFQSASKIITLKK	2.00
K66	-	SKIITLKKRWQLA	0.64
K75	upstream-like	WQLALSKGVHFVC	0.78
K132	-	RLWLCWKCRSKNP	0.73

Threshold was set to 0.3

Table S9. Predicted nitrated Tyr residues of SARS-CoV-ORF3a and SARS CoV-2-ORF3a.

SARS-CoV-ORF3a			SARS-CoV-2-ORF3a		
position	YXXΦ motif	score	position	YXXΦ motif	score
Y91	1st	ΔG=0.111	-	1st	-
Y141	-	ΔG=0.492	-	-	-
Y145	-	ΔG=0.025	Y145	-	ΔG=0.597
-	-	-	Y154	-	ΔG=2.703
Y263	-	ΔG=0.273	Y264	-	ΔG=0.606

ΔG: Free energy in kcal/mol.

Table S10. Predicted S-nitrosylated cysteines (in red) of SARS-CoV-ORF3a and SARS-CoV-2-ORF3a.

SARS-CoV-ORF3a			SARS-CoV-2-ORF3a		
Position	FRP	sequence	Position	FRP	sequence
-	-	-	C200	3.77%	WESGVKDCVVLHSYF

FPR: false positive rate.

Table S11. Predicted S-glutathionylated Cys residues (in red) of SARS-CoV-ORF3a and SARS-CoV-2-ORF3a.

SARS-CoV-ORF3a			SARS-CoV-2-ORF3a		
Position	FRP	sequence	Position	FRP	sequence
-	-	-	C130	4.67%	IIMRLWLCWKCRSKN
-	-	-	C200	0.61%	WESGVKDCVVLHSYF

FPR: false positive rate.

Table S12. Predicted sulfated Tyr residues (in red) of SARS-CoV-ORF3a and SARS-CoV-2-ORF3a.

SARS-CoV-ORF3a			
Y position	YXXΦ motif	sequence	score
154	-	VCWHTHNYDYCIPYN	1.401
156	-	WHTHNYDYCIPYNSV	1.366
184	-	TPKLKEDYQIGGYSE	1.688
200	extra	RHSGVKDYVVVHGYF	0.738
211	4th	HGYFTEVYYQLESTQ	0.594
212	4th	GYFTEVYYQLESTQI	0.485
263	-	NPAMDPIYDEPTTTT	1.450
**SARS-CoV-2-ORF3a**			
Y position	YXXΦ motif	sequence	score
156	-	WHTNCYDYCIPYNSV	1.406
184	-	SPISEHDYQIGGYTE	2.025
211	-	HSYFTSDYYQLYSTQ	1.767
212	-	SYFTSDYYQLYSTQL	0.980
233	4th	EHVTFFIYNKIVDEP	1.248
264	-	NPVMEPIYDEPTTTT	1.218

Threshold was set at 1.139
